# PCOPGene-Net: Holistic Characterisation of cellular states from microarray data based on continuous and non-continuous analysis of gene-expression relationships

**DOI:** 10.1186/1471-2105-10-138

**Published:** 2009-05-09

**Authors:** Mario Huerta, Juan Cedano, Dario Peña, Antonio Rodriguez, Enrique Querol

**Affiliations:** 1 Institut de Biotecnologia i Biomedicina and Departament de Bioquímica i Biología Molecular, Universitat Autònoma de Barcelona, 08193 Bellaterra, Barcelona, Spain

## Abstract

**Background:**

Microarray technology is so expensive and powerful that it is essential to extract maximum value from microarray data, specially from large-sample-series microarrays. Our web tools attempt to respond to these researchers' needs by facilitating the possibility to test and formulate from a hypothesis to entire models under a holistic point of view.

**Results:**

PCOPGene-Net is a web application for facilitating the study of the relationships among gene expressions under microarray conditions, to classify these conditions and to study their effect on expression relationships. Furthermore, the system guides the researcher in the navigation through the microarray data by providing the most suitable genes and information for the researcher's interests at each moment. We achieve all of these by means of the zoom-out operation, the zoom-in operation, the non-continuous analysis and crossing the PCOPGene results with external data-servers.

**Conclusion:**

PCOPGene-Net helps to identify cellular states and the genes involved in these. All of that is accomplished in a flexible way, guided by the researcher's interests and taking advantage of the ability of our system to relate gene expressions, even when these relationships are non-continuous and cannot be found using linear or non-linear analytical methods. Currently, our tools are used for tumour-progression study from a holistic point of view.

## Background

Current biological research, and specially transcriptomics, generates large amounts of data which researchers need to analyse in order to understand the underlying process. DNA microarray technology power lies in its ability to provide data of a great number of gene expressions throughout different sample conditions. There are several relevant web applications for microarray analysis, i.e., GEO [1], BIOREL [2], ArrayExpress [3], MicroGen [4] and GEPAS [5]. Currently, most tools try to extract biological information from such high-throughput expression data combining information from co-expressed genes [6] as well as additional annotations extracted from Gene Ontology (ADGO) [7], phylogenetic information (CLANS)[8] or pathway data (MAPMAN) [9]. In this paper, a new approach incorporating the detailed analysis of the experiments' effect on the fluctuations of gene-expression relationships to the network analysis is proposed.

The microarray data sets can be provided by: **a) **temporal series, useful to study synchronous cellular events, and **b) **serial analysis of gene-expression samples under different conditions (i.e., chemotherapy, temperature, radiation, starvation, etc.) which are more useful for studying asynchronous events. The progressive increase of microarray sample-series [10] motivates a more thorough analysis of expression relationships and gene dependencies throughout these large series, trying to extract global gene behaviours, cellular states and phenotypes. Furthermore, the large number of microarray genes involves genes belonging to very different processes and functions, thus leading to a holistic perspective.

Our strategy to achieve this holistic perspective is to develop several tools which facilitate a progressive analysis of microarrays data with large sample series, leading the researcher from known marker genes, and progressively widen his/her scope analysis towards the holistic perspective. This progressive analysis is based on four approaches:

1. Analysis of linear gene-expression relationships.

2. Analysis of non-linear gene-expression relationships and study of the fluctuations of this non-linearity.

3. Analysis of non-continuous gene-expression relationships.

4. Facilitating the navigation through the microarray data based on: researcher interests, the continuous and non-continuous relationships among gene expressions, and the links among genes supplied by external biomedical databases.

In this way, the tools to enable the progressive analysis are provided.

For this purpose, several methods and tools have been designed [11-13] and integrated in to the four basic operations of the PCOPGene-Net detailed below.

## Methods

### PCOP

The mathematics behind this system makes use of the Principal Curves of Oriented-Points calculation (PCOP) [14,15]. The Principal Curves is a non-linear and non-hypothesis-driven pattern-analysis technique.

PCOP is defined by the generalisation, at the local level, of the next principal-component property, which is: for a normal multivariable distribution *X*, if *X *is projected over the hyperplane, the total variance of the projection is minimised when the hyperplane is orthogonal to the first principal component. In this way, the Principal Oriented Points (POP) are found for each local area, and these local principal-components vectors will be the tangent vectors of the PCOP, the curve which goes through all of the POP. In a previous work [14], it was described how to accurately define these local areas in the global space and the samples belonging to each one. One of the main advantages of PCOP over other methods is the generalisation of the principal-components variance. The PCOP method provides a good measure of the data dispersion around the curve and the capability of a good definition of the local areas, both being very significant for our applications [14].

### Clustering the samples of each POP (the *'hidden-variable-dependent' clustering*)

Using the '*hidden-variable-dependent' clustering operation *[12], the user can select the different discretised states (the POPs) obtained in the PCOP calculus. With this action, the samples belonging to the POP local area are selected. In this way, the user can separate the sample data in term of their belonging to the different local behaviours of gene expression relationships. This new clustering approach, then, permits one to differentiate the samples belonging to a continuous dataset on the basis of the non-explicit reason (or hidden-variable role) of these local tendencies of expression relationships. For more details about this clustering method based on the fluctuations of the inner pattern, consult our work [12].

**Figure 1:**
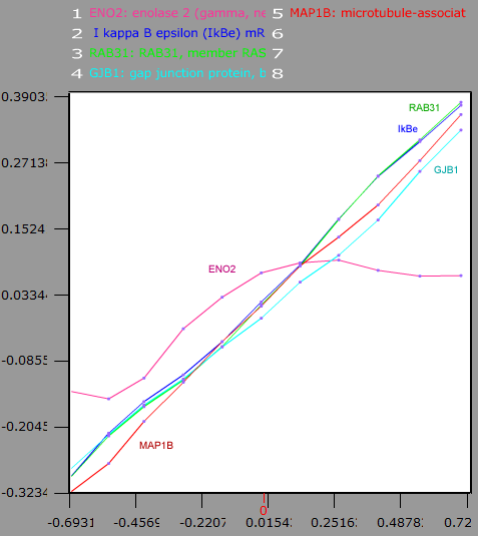
**The Detailed View (parametric plot)**. In the display is shown the dependence relationship among the selected genes. The ordinate axis indicates the expression level while the abscissa indicates the parameter of the function that describes the relationship. The lines represent the expression level of the compared genes for each point of the relationship. Selecting any point of the relationship, a sample class is defined with the samples belonging to this relationship stretch. In the display, it is shown that ENO2 has an expression phase and a non-expression phase, with respect to the rest of the analysed genes.

**Figure 2:**
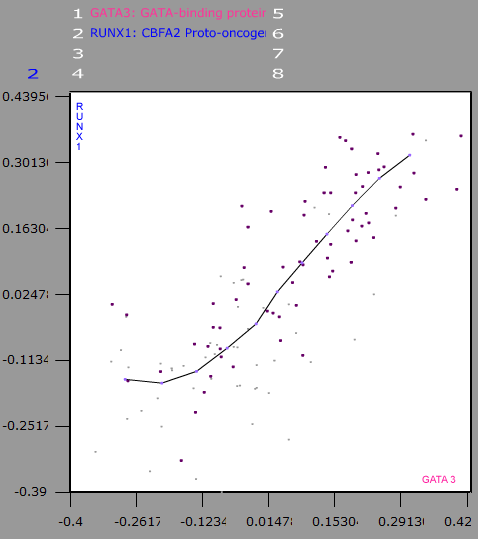
**The Detailed View (geometric plot)**. The expressions of two genes are being compared in the display. Each axis represents the expression level of each gene, the data cloud represents the values of the microarray experiments [32] for the compared genes, and the line represents the dependence relationship between the gene expressions. The samples belonging to different sample classes previously defined are painted with different colours.

In the same interface where the PCOP is shown (*see detailed view*, Fig. 1 and 2), the samples can be clustered to define the classes. Previously-defined classes can be coloured in the same interface to study their influence on gene-expression relationships (Fig. 2).

### Gene network, gene clusters, minimum-spanning tree, and graph layout

The PCOP calculus provides two variance values: the Generalized Total Variance (GTV) and the Residual Variance (RV). The Generalized Total Variance (GTV) is the variance explained by the curve and permits one to know if the Principal Curve is able to follow the sample-cloud tendency. The Residual Variance (*RV*) is the variance not explained by the Curve and describes the degree of dispersion of the samples around the Principal Curve. The Generalized Total Variance (*GTV*) is the sum of these two dispersion parameters, and the **f **factor is the *RV *divided by the *GTV *[15].

This **f **value supplied by the PCOP calculus provides the non-correlation factor and is used to know how many, not exclusively linearly, sampled gene-expressions are related. Once a microarray is uploaded to the server, the PCOP between each pair of microarray genes is calculated. Based on this, a minimum-spanning tree and a complete graph is defined where its edges are the relationships between each pair of genes for the corresponding **f **value. In a previous work [13] is shown the benefit of using the **f **value versus other correlation measurements to construct the gene network.

**Figure 3:**
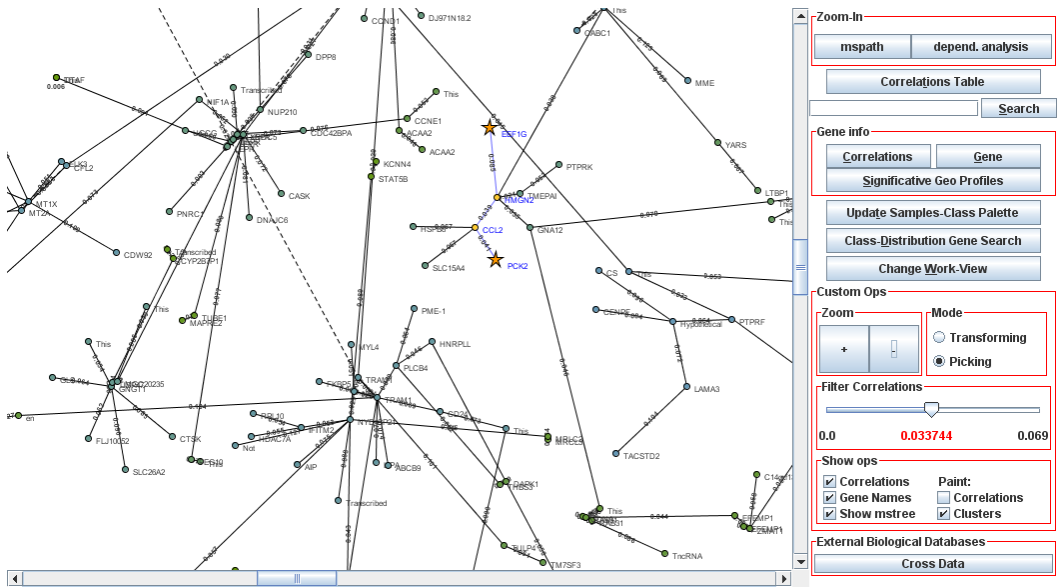
**The Global-Network View**. Interactive network showing the microarray-genes interdependence in expression terms. All of the operations of the PCOPGene-Net are launched from this interface.

The Java JUNG libraries for analysis and visualization of network data by web [16] have been used to mount the interactive graph of the *global vision *interface (Fig. 3). On the graph layout, the genes are placed in the 2D space based on the correlation degree of each gene with its neighbours, grouping the genes in clusters and facilitating the showing of the minimum-spanning path among any set of microarray genes. The minimum-spanning path is necessary for the *zoom-in *operation, as it will be immediately following detailed.

### Gene selection algorithm using the minimum-spanning path

Considering that a query set of microarray genes introduced by the researcher maintains a certain level of correlation as a set (correlation level which the researcher does not wish to lose), the researcher expects to obtain the maximum number of microarray genes which connect the genes of the query set in expression terms, but conserving the correlation level of the query set in the new query-plus-selected-genes set.

The selection algorithm selects the genes (nodes) of the minimum-spanning tree which connect the query genes, conforming the *minimum-spanning path *among them. When a hierarchical clustering is built using a single linkage, we obtain a minimum-spanning tree where each edge of the tree represents the relationship used to add each new gene or cluster to the tree [17]. In this way, we can apply these clusters, their hierarchy and the properties of the hierarchical clustering to their corresponding minimum-spanning tree. And that involves that the minimum-spanning path always converges towards the pair of genes and clusters best correlated among them, maximising the correlation degree of the supplied set of genes [13].

The *gene selection algorithm using the minimum-spanning path *will be used in the *zoom-in *operation explained in the next section.

## Results

The web application is composed of two main graphical interfaces: *The global network view *(Fig. 3) and *the detailed view *(Fig. 1 and 2), some PHPs and CGIs and several databases. The final application provides four basic operations:

1. the zoom-out operation gives a global vision of the interdependence among analysed-microarray gene expressions.

2. At a given moment, the researcher wants to focus his/her research on a concrete, process or on apparently unconnected processes, to know what genes are involved in it and to study their expressions dependence in detail. This task is provided by the *zoom-in *operation.

3. However, a lot of genes are not continuously correlated for all of their expression ranges. To analyse this kind of non-continuous relationships, *the non-continuous analysis *is provided.

4. The number of samples of the analysed microarray are limited and in addition, there are many more links among genes besides the expression links. Remote data-bases will be consulted to elaborate on the information.

**Figure 4:**
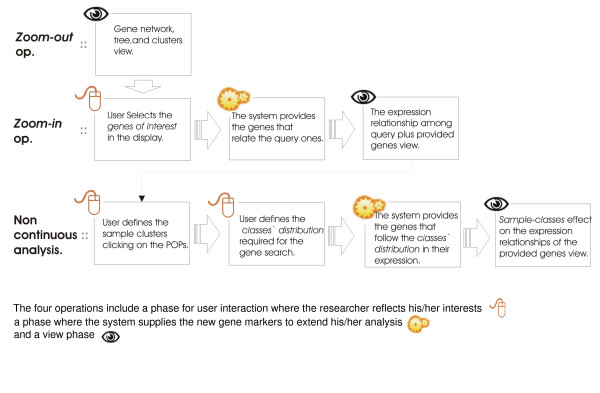
**Basic analysis procedure using the PCOPGene tools: *Zoom-out *operation, *Zoom-in *operation and the non-continuous analysis**.

The four operations are illustrated in Fig. 4 and explained in detail below.

### *Zoom-out *operation

The provides a global network vision with: The graph of continuous relationships among gene expressions (for all of the expression range), the gene clusters with the nodes coloured by the average correlation degree among the cluster genes, the genes most correlated with a selected gene, and the correlation degree of the most correlated genes of a selected gene with the rest of the genes.

### *Zoom-in *operation

The researcher begins selecting his/her genes of interest from the microarray genes, usually gene markers of concrete processes or pathologies that the researcher wants to relate.

Then, the system supplies the genes which link these gene markers in expression terms and throughout the expression range of the genes (continuously). As was seen in the Methods section (*Gene selection algorithm using the minimum-spanning path*), the correlation degree of the *query *set is conserved in the new *query-plus-supplied-genes *set. This is relevant for the final-view step.

Finally, in the *detailed view*, the dependence among the expressions of the *query-plus-supplied-genes *set can be analysed. The fluctuations of this mentioned dependence are shown in the graphical interface (Fig. 1).

### Non-continuous analysis

There are a lot of non-continuous gene-expression relationships impossible to analyse with linear or not linear methods. But furthermore, it can be interesting for the researcher to study only part of a relationship (for instance, from a x = y^2 ^relation, only the region where both genes are underexpressed), it starts a *non-continuous analysis*.

#### First step: Define the classes

The sample classes can be defined from three ways:

Clustering the samples from a part of a gene-expression range.

Clustering the samples from a part of a gene-expression relationship (the *hidden-variable-dependent clustering*, see methods section).

Sample clustering based on previous knowledge: The clusters comes from *a) *medical or biological origin, provided for example by the microarray developers, or *b) *obtained by different statistical methods such as k-nearest neighbor [18], Self-organizing Maps [19], Principal Components, Biclustering [20], Locally Linear Embedding [21] and so on.

The advantage of defining the clusters from an expression relationship instead of an expression range is that we are selecting the samples by the tendency maintained by the relationship among two or more genes. As a result, our *clustering from an expression relationship *class definition more accurately defines the boundaries of the cell state that we wish to associate with the sample class [11]. In contrast to the *previous knowledge *way using a statistical method that clusters the sample conditions by gene-expression similarity using all of the genes (or some of them in Biclustering [22]), in the *clustering from an expression relationship *way, the sample clustering uses only the gene markers of the processes in which the researcher is interested (and based on the tendency of their expression relationship). One of the main features that distinguishes our cluster and class definition tools from the global statistic methods used in the current software or the sample classes defined by the microarray authors used in GEO [1] is the flexibility of our progressive class definitions.

#### Second step: Searching non-continuous relationships

Once the sample classes are defined, the next step is to define a class distribution and search these genes that follow this distribution of classes along their expressions. For instance, we can query the genes in whose expressions one class is over-expressed, with respect another. The supplied genes should maintain a non-continuous relationships among their expressions, where these relationships will be defined by the class distribution required in the search [11].

#### Last step: Observing the classes in the detailed view

Finally, in *the detailed view*, the distribution of the sample classes along a gene-expression relationship can be observed (Fig 2). In this way, the effect of each class on the fluctuations of the dependence relationship can be analysed.

### Biomedical Database Remote access

The purpose is to supply complementary information beyond expression relationships.

The GEO Microarrays-database [1] would be accessed attempting to know if the marker genes found for the analysed microarray (for instance, the genes supplied by the search of the last section) are gene markers in any GEO datasets.

The searches of the *zoom-in *operation and non-continuous analysis select new gene markers in expression terms. However, the researcher cannot know much more about these genes at first. Accessing the remote databases, the researcher can contextualise these relationships with new biological and medical information; for instance, observing their location in KEGG maps [23], their proteins' interaction [24-26], PubMed [27] papers that talk about their connection, etc.

There are gene relationships that cannot be found in expression terms. Nevertheless, these relationships can be found accessing the remote biomedical databases (for instance, interaction at the protein level, activation by means of phosphorylation, proteolytic inactivation, etc.). In this way the researcher can extend his/her analysis to new genes related with the current genes of interest but not exclusively in expression terms. The scope of this new-genes search can be limited to the clusters of selected genes or it can be limited by the level of correlation with these selected genes.

### Assigning attributes to the sample classes

Our main objective is the holistic study of cell behaviour taking advantage of the high-throughput potential of microarray data. The classes definition and the attributes assignment to sample classes are the key points to achieve this. In this way, the cellular states and processes should be characterised, trying to associate them with a concrete class or subclass.

The attributes should give biological meaning to the sample classes, helping to identify and describe the cell state in its widest definition, which each class represents. The assignment of attributes comes from the four operations previously described: From the non-continuous analysis comes the definition of sample classes, from the *zoom-in *operation and non-continuous analysis comes the identification of gene markers of each class, and from the access to remote databases come the attributes assigned to each class on the basis of the gene markers of each one. It works as follows.

#### Obtaining gene markers from continuous analysis

The dependence relationship among gene expressions can be observed in continuous analysis (Fig. 1 and 2). Each fluctuation of this dependence can be selected as a new sample class. Then, the compared genes become gene markers of the new sample class, giving new attribute to it s. Note that the compared genes can be gene markers of apparently unconnected processes.

#### Obtaining gene markers form non-continuous analysis

The genes supplied by the search will be the gene markers for the classes used as search parameters. These gene markers will give new attributes to the classes.

#### Defining subclasses and obtaining more gene markers

When a subclass is defined from a sample class selecting only a part of the class samples, the subclass has got the attributes of the original class plus the new and differentiated attributes. It is commonly performed when a previously defined class is observed in a new relationship analysis, and the researcher wishes to define a new class from this relationship while conserving the original class discrimination (Fig. 2). The genes compared in the new relationship analysis will be gene markers of the new subclass but not of the original one.

#### Obtaining attributes based on gene markers by accessing remote databases

The attributes can come from two kinds of databases: Ones about gene-expressions and others besides gene-expressions.

Accessing the GEO microarray datasets [1] for class gene-marker, the researcher obtains the microarrays for which this gene is also a gene marker. The attributes of these microarray sample series will be the new attributes of the class.

Accessing the biomedical databases [1,23-31] for class gene-markers, the researcher will obtain the attributes that will give the biological and medical significance to sample classes. Notice that in this way the information will not be supplied for each gene separately but rather for all of the gene markers as a whole, defining the cell state of the sample class.

## Conclusion

Let us see now how the four described operations are adjusted to the four approaches followed to achieve the progressive analysis towards the holistic perspective.

Through the *zoom-in *and *zoom-out *operations, the analysis of the linear and non-linear relationships among gene-expressions are made. With the *zoom-in *operation, the detailed analysis of the expression-dependence fluctuations is also provided.

Through the non-continuous analysis, the non-continuous relationships among gene-expressions that cannot be detected by linear and non-linear methods can be finally analysed.

The progressive extension of the analysis scope based on researcher's interests are present in all four operations: In the *zoom-in *operation, the genes linking the query ones are supplied. In the non-continuous analysis, the genes whose expression follows the required class distribution are also supplied and the classes are successively redefined in to subclasses. The access to remote databases enables expanding the analysis beyond the expression relationships.

All of the operations are highly flexible and complementary using the results of one as an input of the next one, thus making the progressive analysis possible. The purpose is to obtain the sample classes and attributes that characterise these classes and, in a roundabout way, characterise the cellular state or phenotype that these classes represent.

With this analysis of the microarray sample conditions divided into classes, we deal with the principal objective of our web application: "Studying the cell behaviour and phenotype changes from a holistic point of view". Note that gene markers of very different processes can be related in all of the operations for extending the analysis (taking advantage of the high-throughput capability of microarray technology). The web application is currently used for tumour study from a holistic point of view, covering fields like cellular stress, cell proliferation, tissue remodelling, mitochondrial activity, oxygen and ROS/NOS levels, cell differentiation and dedifferentiation, membrane polarity, intracellular and extracellular pH levels, circadian rhythms, the inference of the sympathetic and parasympathetic nervous system, metastasis, or interaction with viruses and bacteria.

## Availability and requirements

• **Project name: **PCOPGene

• **Project home page: **

• **Operating system(s): **Web-based application

• **Programming language: **PHP, Java, flash-script, CGI, C++, Perl, Matlab (interfaces for matlab users).

• **Other requirements: **Mozilla 5.0, sea monkey 1.0, Firefox 1.0 or Explorer 6.0. flash plug-in 7.0 and Java 1.3.1 or higher versions.

• **License: **Free access. Source codes available in the web.

• **Any restrictions for use by non-academics: **we prefer non-profit use.

The server is freely accessible for guest users. However, users are encouraged to fill in a very simple registration form for better privacy and convenience. A PCOPGene-Net tour is provided to describe the application's use, and it is indexed from the application help. For mathematical and computational details, technical reports are also available in the web.

## Authors' contributions

MH and JC designed the methods and the application, its use on microarray data sets for researching and writing the paper. DP and AR performed the implementation. EQ has supported the process, and is the link with other research teams in the use of the PCOPGene application in their own data-analysis, tackling areas such as neurosciences or tumour-genesis.
